# Comparison of dynamic changes in the peripheral CD8^+^ T cells function and differentiation in ESCC patients treated with radiotherapy combined with anti-PD-1 antibody or concurrent chemoradiotherapy

**DOI:** 10.3389/fimmu.2022.1060695

**Published:** 2022-11-21

**Authors:** Hui Wei, Yanqi Li, Zhoubo Guo, Xiaoxue Ma, Yang Li, Xiaoying Wei, Dong Han, Tian Zhang, Xi Chen, Cihui Yan, Jiahuan Zhou, Qingsong Pang, Ping Wang, Wencheng Zhang

**Affiliations:** ^1^ Department of Radiation Oncology, Tianjin Medical University Cancer Institute and Hospital, National Clinical Research Center for Cancer, Key Laboratory of Cancer Prevention and Therapy, Tianjin’s Clinical Research Center for Cancer, Tianjin, China; ^2^ Department of Immunology, Tianjin Medical University Cancer Institute and Hospital, National Clinical Research Center for Cancer, Key Laboratory of Cancer Prevention and Therapy, Tianjin’s Clinical Research Center for Cancer, Tianjin, China; ^3^ Department of Clinical Research and Development, Jiangsu Hengrui Pharmaceuticals Co., Ltd, Shanghai, China

**Keywords:** CD8+ T cells, PD-1, radiotherapy, immunotherapy, chemoradiotherapy, ESCC

## Abstract

**Objective:**

The systematic immune status of cancer patients undergoing immunotherapy is little known. We prospectively identified the function and differentiation traits of peripheral CD8^+^ T cells based on our phase 1b clinical trial (NCT03222440) of radiotherapy combined with camrelizumab in patients with locally advanced esophageal squamous cell carcinoma (ESCC) and compared it with concurrent chemoradiotherapy (CCRT).

**Methods:**

19 and 18 patients were included in the cohort of radiotherapy plus camrelizumab and cohort of CCRT treatment. By using flow cytometry, we evaluated the expression levels of PD-1, Eomes, T-bet and IFN-γ (function), CD38 and HLA-DR (activation), and differentiation subsets classified according to the expression levels of CD45RA and CD62L in peripheral CD8^+^ T cells before and during treatment.

**Results:**

Effective binding of anti-PD-1 antibody camrelizumab with PD-1 on CD8^+^ T cells was detected during treatment. Both two treatments elevated the expression levels of activation molecules CD38 and HLA-DR on CD8^+^ T cells. PD-1^+^CD8^+^ T cells had more activation features than PD-1^-^CD8^+^ T cells in two groups and the treatments did not alter these differences. The two treatments activated both PD-1^+^ and PD-1^-^ CD8^+^ T cells. PD-1^+^CD8^+^ T cells had less Naïve and TEMRA but more Tcm and Tem than PD-1^-^CD8^+^ T cells in two groups and both two treatments changed the ratio of memory T cells in PD-1^+^ and PD-1^-^ cells. RT plus camrelizumab treatment reduced Naïve T cells and TEMRA subsets both in PD-1^+^ and PD-1^-^ CD8^+^ T cells while elevated Tcm subset in PD-1^+^CD8^+^ T cells and Tem subset in PD-1^-^CD8^+^ T cells. CCRT elevated Tcm subset and reduced TEMRA subset in PD-1^-^CD8^+^ T cells while did not change any subset in PD-1^+^CD8^+^ T cells. Furthermore, patients undergoing radiotherapy plus immunotherapy were found to obtain better prognosis than those receiving CCRT.

**Conclusions:**

This study identified the dynamic changes of systematic immune status of patients undergoing treatment. The two treatments had similar activation effects on peripheral CD8^+^ T cells with different PD-1 properties but had different effects on their differentiation status. These results provided potential clues to the reasons underlying the difference in prognosis of the two treatments.

## Introduction

The incidence and mortality rates of esophageal cancer are ranked seventh and sixth in the world, respectively (1). More than 90% of esophageal cancer is esophageal squamous cell carcinoma (ESCC). Patients with ESCC are typically diagnosed at the locally advanced stage and concurrent chemoradiotherapy (CCRT) is the standard treatment (2).

As anti-PD-1 antibodies were first used to treat drug-resistant solid tumors in 2006 (3), immune checkpoint blockades have recently been successfully applied in various cancers (4–7). Several studies including researches on glioma, implanted breast and colorectal carcinomas, and non-small cell lung cancer have supported the combination of radiotherapy and immunotherapy (8–11). From July 2017 to January 2018, we firstly conducted a phase 1b clinical trial (NCT03222440) of radiotherapy concurrently combined with an anti-PD-1 antibody, camrelizumab, as first-line treatment in patients with locally advanced ESCC who were intolerant to or refused CCRT. This combination therapy had manageable toxicity and preliminary antitumor efficacy for locally advanced ESCC (12).

The occurrence and development of tumors are closely related to antitumor immune responses. Peripheral blood lymphocytes (PBLs) can directly reflect a patient’s systemic immune status. To date, a few studies focused on PBLs have preliminarily confirmed that the changes of lymphocyte subsets before and after treatment have predictive significance for treatment response (13–15). For example, increased CD8^+^CD28^+^ T cells predict better early response to stereotactic ablative radiotherapy in non-small cell lung cancer and high frequency of PD-1^+^TIGIT^+^CD8^+^ T cells predict better response to anti-PD-1 therapy in melanoma and Merkel cell carcinoma.

CD8^+^ T cells are the main effector T cells that play a vital role in antitumor immunity. Based on CD45RA and CD62L expression, CD8^+^ T cells are divided into four differentiation subtypes: naïve T cells (CD45RA^+^CD62L^+^), central memory T cells (Tcm, CD45RA^-^CD62L^+^), effector memory T cells (Tem, CD45RA^-^CD62L^-^), and CD45RA^+^ effector memory T cells (TEMRA, CD45RA^+^CD62L^-^) (16). Molecular markers can characterize the state of T cells. PD-1 is a checkpoint molecule which regulates immunocytes status (17). Eomes and T-bet are two members of the T-box transcription factor family (18). Both of them regulate T-cell effector functions, including IFN-γ production (19–21). IFN-γ is a crucial cytokine for innate and adaptive immunity and contributes to the antitumor immune response through its immunostimulatory and immunomodulatory effects (22). CD38 and HLA-DR are markers of T cell activation (23, 24). Detecting the expression of these molecule markers could indicate repressed or activated state of T cells.

At present, there are few reports on feature changes of CD8^+^ T cells in the peripheral blood of patients with ESCC under treatment. We previously reported the changes of main T-cell subsets, CD4^+^ and CD8^+^ T cells, under radiotherapy plus immunotherapy (12). In the present study, by further monitoring the expression levels of molecular markers and differentiation subsets in the peripheral CD8^+^ T cells of patients with locally advanced ESCC before and during radiotherapy plus immunotherapy or CCRT, we attempted to determine the dynamic changes of activation, function and differentiation status of peripheral CD8^+^ T cells and compare the similarities and differences between the two cohorts. This may provide evidence for the understanding of systematic immune status of patients undergoing treatment and contribute to the treatment of patients with locally advanced ESCC.

## Materials and methods

### Patients

We described patients treated with RT plus camrelizumab in a former phase 1b clinical trial (NCT03222440, n=19) (12). In brief, we recruited patients with histologically confirmed ESCC who were not acceptable to CCRT at Tianjin Medical University Cancer Institute and Hospital between July 2017 and January 2018. Besides, 18 patients diagnosed with ESCC who received CCRT treatment were included at Tianjin Medical University Cancer Institute and Hospital from August 2017 to May 2018, and these patients were treatment-naïve prior to CCRT. Patients received radiotherapy (2 Gy/fraction, 5 fractions/week, total 60 Gy). Camrelizumab treatment (200 mg every 2 weeks) started with radiotherapy and continued for 32 weeks. The CCRT scheme was radiotherapy combined with Docetaxel and Cisplatin. All patients offered informed consents before being included in the study. This study was approved by the Ethical Committee of Tianjin Medical University Cancer Institute and Hospital.

### Peripheral blood samples collection

EDTA-anticoagulant treated peripheral blood samples were collected on the day before the first dose of RT and after the delivery of 40 Gy RT (during treatment). Peripheral blood samples were centrifugated using Ficoll gradient centrifuge separation (Eurobio Ficoll). And peripheral blood mononuclear cells (PBMCs) were isolated from the buffy coat layer after centrifugation. Purification of PBMCs for flow cytometry analysis was done no more than 2 hours after the fresh whole-blood collection.

### Flow cytometry analysis

PBMCs suspensions were preincubated with Human TruStain FcX™ (Biolegend, cat. 422302) at 4 °C for 5 min to block the FcR-involved unwanted staining. Then, fluorescent dye-conjugated primary antibodies for cell surface markers staining were added directly to the preincubated cells in the presence of Fc Block™. To detect anti-PD1 antibody camrelizumab binding in patients during RT plus immunotherapy treatment, a mouse anti-human IgG4 Fc-PE (SouthernBiotech, cat. 9200-09) was used without prior Fc blocking. Cells were held for 30 min at 4°C in the dark and washed with staining buffer. Next, intracellular markers staining was performed after the cells were fixed and permeabilized by using eBioscience™ Foxp3/Transcription Factor Staining Buffer Set (ThermoFisher, cat. 00-5523-00). After being washed, samples were tested immediately with a BD FACSAira III flow cytometry system. BD GolgiPlug™ (cat. 550583) was used to stimulate PBMCs separated from the fresh peripheral blood to test IFN-γ. CD8, CD45RA, CD62L, PD-1, CD38 and HLA-DR were cell surface markers and Eomes, T-bet and IFN-γ were intracellular markers. CD38 and HLA-DR were tested in panel 1 with Eomes, T-bet and IFN-γ in panel 2. CD8, CD45RA, CD62L and PD-1 were examined in both panels. The following specific fluorochrome-labeled monoclonal antibodies were used: CD45RA-APC-H7 (BD, cat. 560674), CD62L-APC (BD, cat. 559772), CD8-BV510 (BD, cat. 563256), PD-1-PE (Biolegend, cat. 329906), PD-1-BV421 (Biolegend, cat. 329920), Eomes-PE-eFluor™ 610 (eBioscience, cat. 61-4877-42), T-bet-FITC (Biolegend, cat. 644812), IFN-γ-PerCP-Cy™5.5 (BD, cat. 560704), CD38-PE/Dazzle™ 594 (Biolegend, cat. 356630) and HLA-DR-PE/Cy7 (Biolegend, cat. 307616). Data of the two panels were available in 19 patients in RT plus camrelizumab group with 19 baseline and on-treatment pairs, and 18 patients in CCRT group with 14 corresponding pairs. Data were analyzed by using FlowJo software (Version 10.4).

### Statistical analysis

Statistical analyses were conducted using SPSS (Version R26.0.0.0) and GraphPad Prism (Version 9.1.1). Differences in baseline characteristics between patients in RT plus camrelizumab group and CCRT group were analyzed by unpaired Student’s *t*-test and Mann-Whitney test for age and primary tumor diameter as well as Fisher’s exact test for other characteristics. Changes in the expression levels of molecular markers and differentiation subsets before and during treatment, as well as comparison of molecule expression and differentiation subsets in PD-1^+^ and PD-1^-^ CD8^+^ T cells were calculated by paired Student’s *t*-test for the normal parametric test, whereas Wilcoxon test was used for the nonparametric test. Comparison of the increased ratio (during/before treatment) of CD38 and HLA-DR expression levels between RT plus camrelizumab and CCRT treatment was calculated by unpaired Student’s *t*-test for the normal parametric test, whereas Mann-Whitney test was used for the nonparametric test. Survival analysis was performed by plotting Kaplan-Meier survival curves, and log-rank tests were elicited to evaluate the differences. Data were presented as mean (lower 95% CI of mean, upper 95% CI of mean). All statistical tests were two-sided and a *P* value of < 0.05 represented statistical significance.

## Results

### Effects of radiotherapy combined with immunotherapy and CCRT on the levels of molecular markers in peripheral CD8^+^ T cells

**Figure 1:**
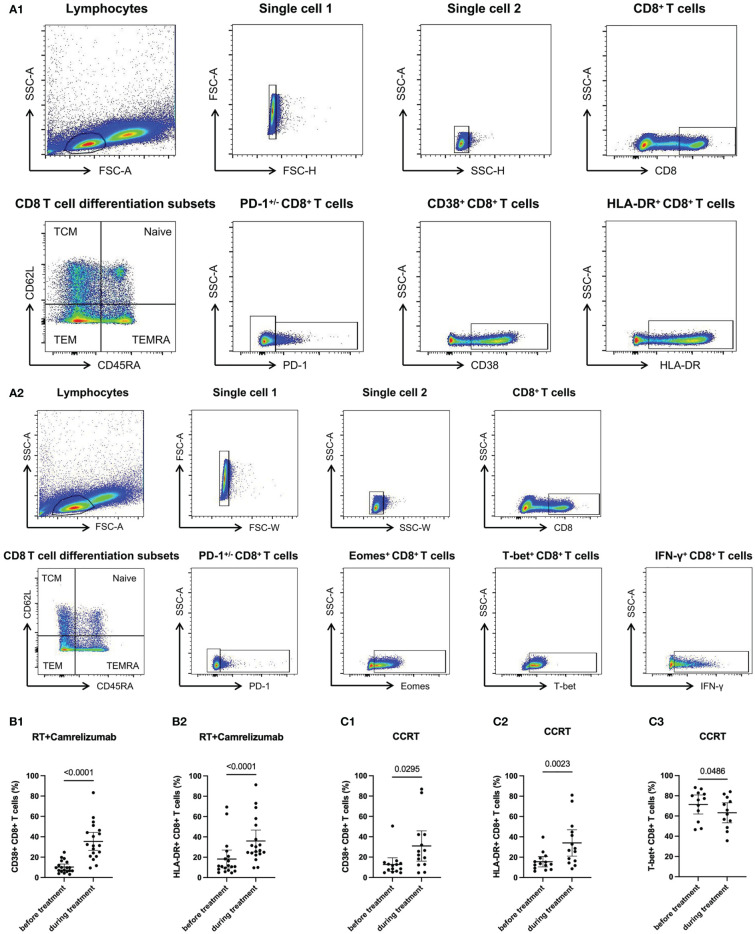
The expression levels of molecular markers in peripheral CD8^+^ T cells. **(A)** Schematics of the data processing of panel 1 and 2. **(B)** CD38 and HLA-DR expressions on CD8^+^ T cells in RT plus camrelizumab group. **(C)** CD38, HLA-DR and T-bet expressions in CD8^+^ T cells in CCRT group.

Data of 19 patients in RT plus camrelizumab group and 18 patients in CCRT group were included in our study. Schematics of the data processing from flow cytometry were shown in **Figure 1A**. There was no difference in baseline characteristics between the two groups (**Table 1**). We firstly detected the expression levels of several molecular markers, including checkpoint molecular PD-1, functional molecules (Eomes, T-bet and IFN-γ), and activated molecules (CD38 and HLA-DR) in peripheral CD8^+^ T cells both before and during RT plus camrelizumab or CCRT treatment. Effective binding of anti-PD-1 antibody camrelizumab with PD-1 on CD8^+^ T cells was detected during RT plus camrelizumab treatment and the proportion of camrelizumab binding CD8^+^ T cells was 10.48% (6.26%, 14.69%). The proportions of CD38 and HLA-DR on CD8^+^ T cells elevated significantly after RT plus camrelizumab treatment (**Figure 1B**). RT plus camrelizumab did not affect the expression levels of Eomes, T-bet and IFN-γ in CD8^+^ T cells (**Figure S1A**). CCRT significantly reduced T-bet level in CD8^+^ T cells, while elevated CD38 and HLA-DR levels on CD8^+^ T cells (**Figure 1C**). CCRT had no effects on the expression levels of PD-1, Eomes and IFN-γ in CD8^+^ T cells (**Figure S1B**).

**Table 1:** Baseline characteristics of patients in two groups.

	RT plus camrelizumab group (n = 19)	CCRT group (n = 18)	*P* value
Age (y)	63 (59, 67)	59 (56, 61)	0.10
Gender, n (%)			0.27
Female	7 (37%)	3 (17%)	
Male	12 (63%)	15 (83%)	
ECOG performance status, n (%)			0.75
0	10 (53%)	8 (44%)	
1	9 (47%)	10 (56%)	
Primary tumor diameter (cm)	5.11 (4.26, 5.95)	5.72 (4.52, 6.93)	0.38
Primary tumor location, n (%)			0.54
Cervical segment	1 (5%)	4 (22%)	
Upper thoracic segment	5 (26%)	4 (22%)	
Middle thoracic segment	11 (58%)	8 (44%)	
Inferior thoracic segment	2 (11%)	2 (11%)	
AJCC8 disease stage, n (%)			0.69
I	0 (0%)	1 (6%)	
II	1 (5%)	1 (6%)	
III	11 (58%)	7 (39%)	
IV	7 (37%)	9 (50%)	
Smoking status, n (%)			0.30
Never	8 (42%)	4 (22%)	
Former or current	11 (58%)	14 (78%)	
Drinking status, n (%)			0.17
Never	9 (47%)	4 (22%)	
Former or current	10 (53%)	14 (78%)	

Date of age and primary tumor diameter were shown as mean (lower 95% CI of mean, upper 95% CI of mean).

### Different functional and activated characteristics between PD-1^+^ and PD-1^-^ CD8^+^ T cells

**Figure 2:**
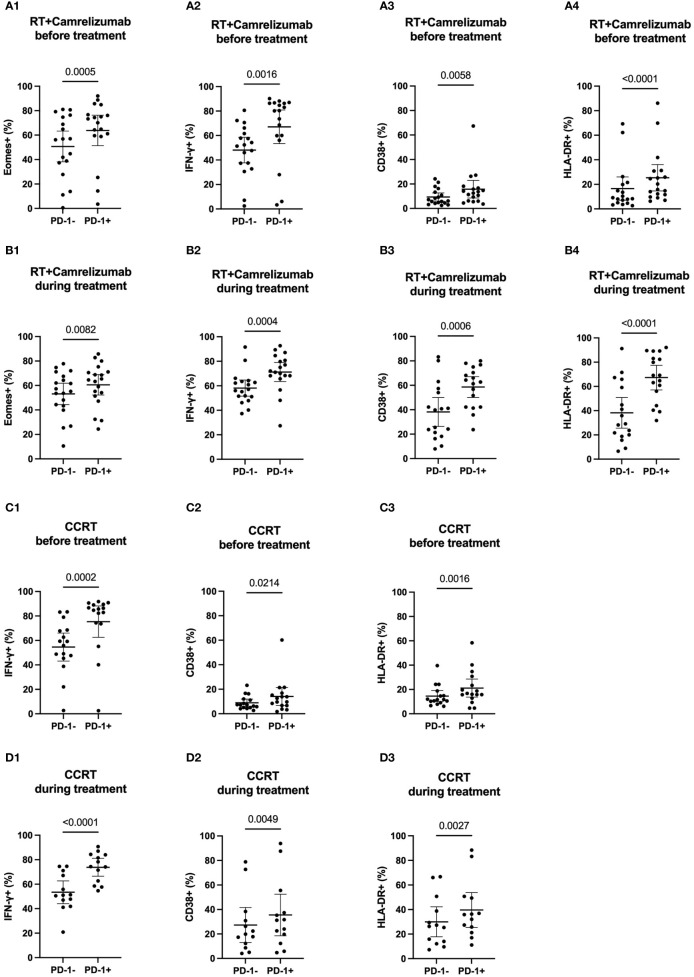
The expression levels of molecular markers in PD-1^+^ and PD-1^-^ CD8^+^ T cells. **(A)** before RT plus immunotherapy treatment. **(B)** during RT plus immunotherapy treatment. **(C)** before CCRT treatment. **(D)** during CCRT treatment.

We divided the peripheral CD8^+^ T cells into PD-1^+^ and PD-1^-^ subsets to explore the different expression levels of functional and activated molecules between the two subpopulations before and during radiotherapy plus immunotherapy or CCRT treatment (**Figure S2A**). The expression levels of Eomes, T-bet, IFN-γ, CD38 and HLA-DR in PD-1^+^ and PD-1^-^ CD8^+^ T cells were shown in **Table 2**. PD1^+^CD8^+^ T cells had higher expression levels of Eomes, IFN-γ, CD38 and HLA-DR compared with PD1^-^CD8^+^ T cells both before and during RT plus camrelizumab treatment (**Figures 2A, B**). Whereas, there was no significant difference in the expression level of T-bet between PD-1^+^ and PD-1^-^ CD8^+^ T cells either before or during RT plus camrelizumab treatment (**Figures S2B, C**). We observed similar results in CCRT group except for Eomes which showed equal levels between PD-1^+^ and PD-1^-^ CD8^+^ T cells both before and during CCRT treatment (**Figures 2C, D**; **Figures S2D, E**). These results indicated that the peripheral PD1^+^CD8^+^ T cells exhibited higher functional and activated characteristics compared with PD1^-^CD8^+^ T cells, and neither RT plus camrelizumab nor CCRT treatment significantly influenced these differences between the two T-cell subsets.

**Table 2:** The expression levels of molecule markers in PD-1^+^ and PD-1^-^ CD8^+^ T cells.

	RT plus camrelizumab group			CCRT group		
	Before treatment	During treatment	*P* value	Before treatment	During treatment	*P* value
PD-1^+^CD8^+^ T cells (%)						
Eomes	63.83 (51.39, 76.27)	60.12 (51.31, 68.93)	0.12	58.95 (44.58, 73.33)	47.05 (33.81, 60.29)	0.08
T-bet	60.47 (45.29, 75.65)	66.86 (56.48, 77.24)	0.36	73.41 (60.86, 85.96)	66.16 (50.26, 82.07)	0.42
IFN-γ	69.46 (55.75, 83.16)	69.96 (62.17, 77.76)	0.78	72.77 (55.45, 90.09)	73.43 (65.47, 81.38)	0.62
CD38	15.15 (7.58, 22.72)	58.61 (50.08, 67.13)	<0.001	10.99 (7.00, 14.98)	35.82 (16.20, 55.44)	0.01
HLA-DR	25.34 (13.89, 36.79)	67.28 (57.15, 77.40)	<0.001	18.70 (11.42, 25.98)	40.31 (23.68, 56.94)	<0.01
PD-1^-^CD8^+^ T cells (%)						
Eomes	50.68 (38.08, 63.27)	53.60 (44.39, 62.81)	0.66	52.67 (38.17, 67.18)	44.81 (30.38, 59.23)	0.17
T-bet	62.61 (49.09, 76.12)	71.90 (62.69, 81.11)	0.15	77.67 (68.38, 86.97)	72.22 (60.56, 83.87)	0.50
IFN-γ	47.58 (36.57, 58.60)	56.16 (50.75, 61.57)	0.17	52.32 (37.81, 66.83)	53.61 (43.42, 63.79)	0.87
CD38	8.94 (5.54, 12.33)	38.21 (26.37, 50.05)	<0.001	8.52 (5.48, 11.55)	27.65 (10.68, 44.62)	0.02
HLA-DR	16.72 (6.60, 26.85)	38.20 (25.50, 50.90)	<0.001	14.80 (8.16, 21.44)	30.61 (16.27, 44.96)	0.01

Paired data available at baseline and on-treatment were included. Date was shown as mean (lower 95% CI of mean, upper 95% CI of mean).

### Dynamic characteristics in PD-1^+^ and PD-1^-^ CD8^+^ T-cell subsets under radiotherapy plus immunotherapy or CCRT treatment

**Figure 3:**
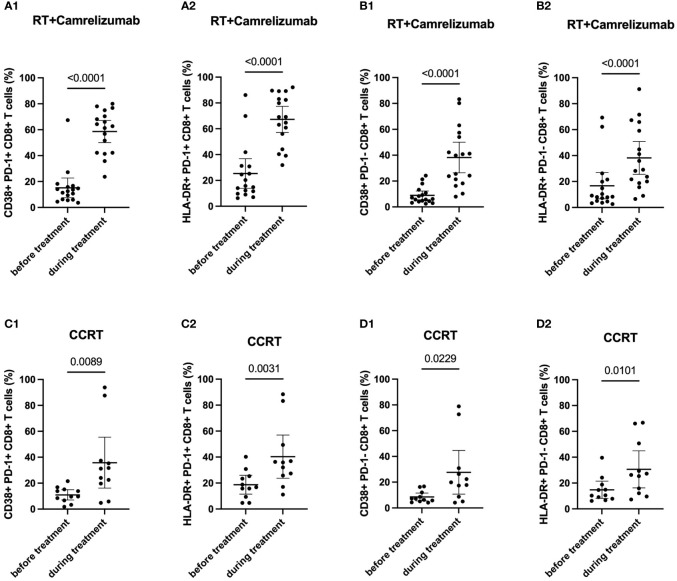
Dynamic characteristics in PD-1^+^ and PD-1^-^ CD8^+^ T-cell subsets. **(A)** PD-1^+^CD8^+^ T cells in RT plus immunotherapy group. **(B)** PD-1^-^CD8^+^ T cells in RT plus immunotherapy group. **(C)** PD1^+^CD8^+^ T cells in CCRT group. **(D)** PD1^-^CD8^+^ T cells in CCRT group.

To evaluate the effects of treatment on PD-1^+^ and PD-1^-^ CD8^+^ T cells, we then examined the dynamic expression levels of functional and activated molecules on these two subsets (**Table 2**). We found that the expression levels of CD38 and HLA-DR elevated significantly while Eomes, T-bet and IFN-γ did not change in both PD-1^+^ and PD-1^-^ CD8^+^ T cells after RT plus camrelizumab treatment (**Figures 3A, B**; **Figures S3A, B**). The similar findings were also observed after CCRT treatment (**Figures 3C, D**; **Figures S3C, D**). Additionally, we compared the increased ratio (during/before treatment) of CD38 and HLA-DR expression levels on PD-1^+^CD8^+^ T cells as well as PD-1^-^CD8^+^ T cells between RT plus camrelizumab and CCRT treatment and did not find differences between the two groups (**Table 3**). These results suggested that both RT plus camrelizumab and CCRT treatment had similar effects on activation characteristics in PD-1^+^ and PD-1^-^ CD8^+^ T-cell subsets.

**Table 3:** The increased ratio (during/before treatment) of CD38 and HLA-DR expression levels on PD-1^+^ and PD-1^-^ CD8^+^ T cells.

	RT plus camrelizumab group	CCRT group	*P* value
PD-1^+^CD8^+^ T cells (the increased ratio)			
CD38	5.70 (3.94, 7.46)	3.92 (1.58, 6.25)	0.08
HLA-DR	3.93 (2.78, 5.08)	2.53 (1.47, 3.60)	0.05
PD-1^-^CD8^+^ T cells (the increased ratio)			
CD38	4.92 (3.71, 6.13)	3.39 (1.59, 5.19)	0.12
HLA-DR	4.11 (1.54, 6.67)	2.19 (1.48, 2.90)	0.23

Date was shown as mean (lower 95% CI of mean, upper 95% CI of mean).

### Differentiation status in PD-1^+^ and PD-1^-^ CD8^+^ T cells

**Figure 4:**
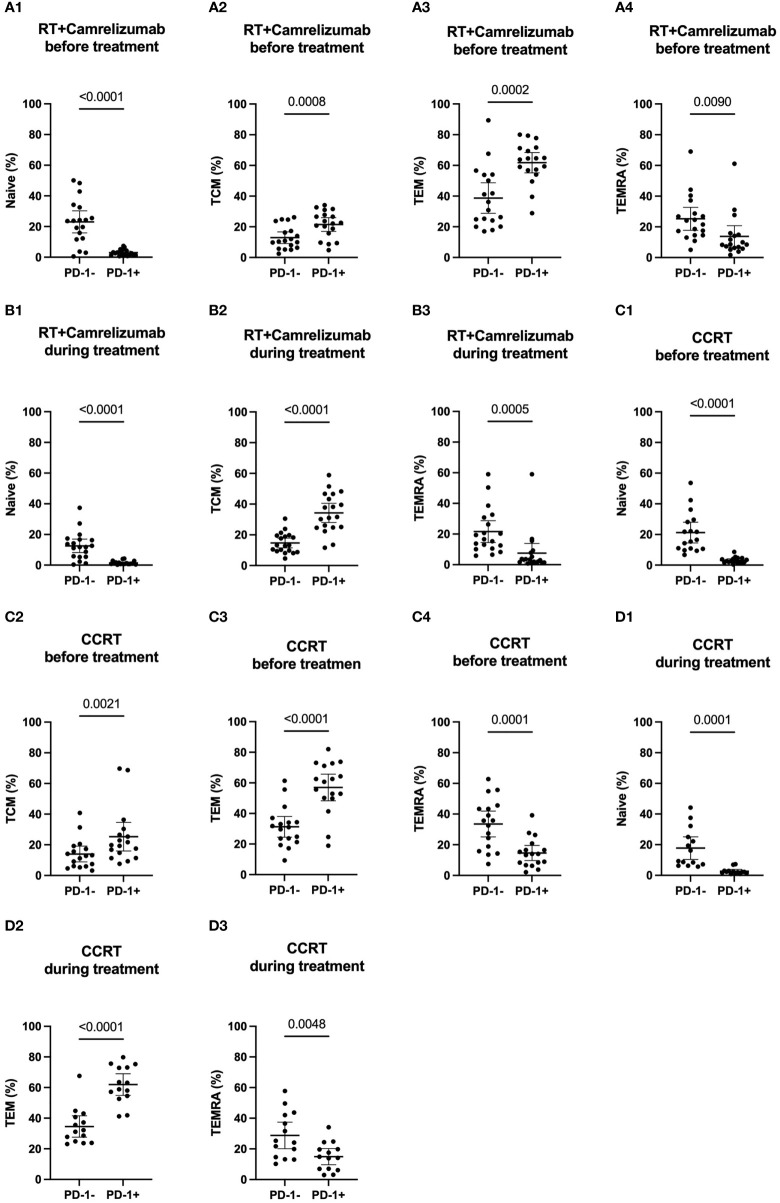
Differentiation subsets of PD-1^+^ and PD-1^-^ CD8^+^ T cells. **(A)** before RT plus immunotherapy treatment. **(B)** during RT plus immunotherapy treatment. **(C)** before CCRT treatment. **(D)** during CCRT treatment.

To further identify the different characteristics of PD-1^+^ and PD-1^-^ CD8^+^ T cells, we next analyzed the differentiation status in these two T-cell subsets (**Figure S2A**). Tem subset was the most subset in both PD-1^+^ and PD-1^-^ CD8^+^ T cells and both before and during the two groups, except that TEMRA subset was slightly more than Tem subset in PD-1^-^CD8^+^ T cells before CCRT treatment (**Table 4**). PD-1^+^CD8^+^ T cells had less Naïve T cells and TEMRA subsets than PD-1^-^CD8^+^ T cells both before and during RT plus camrelizumab or CCRT treatment. Contrastively, PD-1^+^CD8^+^ T cells had more Tcm and Tem subsets compared with PD-1^-^CD8^+^ T cells before two treatments. However, the distinctive proportions of Tem and Tcm between PD-1^+^ and PD-1^-^ CD8^+^ T cells were lost after RT plus camrelizumab and CCRT treatment, respectively (**Figures 4**, **Figure S4**). These results revealed that the peripheral PD-1^+^CD8^+^ T cells had more memory properties than PD-1^-^CD8^+^ T cells before treatment, and the differentiation status of these two subsets might be affected by RT plus camrelizumab and CCRT treatment.

**Table 4:** Differentiation status in PD-1^+^ and PD-1^-^ CD8^+^ T cells.

	RT plus camrelizumab group				CCRT group		
	Before treatment	During treatment		*P* value	Before treatment	During treatment	*P* value
PD-1^+^CD8^+^ T cells (%)							
Naive	2.92 (1.90, 3.94)	1.41 (0.83, 1.98)	<0.001		2.51 (1.79, 3.23)	2.74 (1.49, 3.98)	0.89
Tcm	21.50 (17.08, 25.93)	34.83 (28.33, 41.33)	<0.001		22.52 (13.18, 31.86)	20.47 (16.06, 24.87)	0.64
Tem	61.76 (55.13, 68.38)	56.78 (49.63, 63.93)	0.10		59.38 (50.16, 68.60)	60.95 (53.62, 68.27)	0.76
TEMRA	13.83 (6.83, 20.83)	6.98 (0.27, 13.70)	<0.001		15.61 (9.17, 22.04)	15.87 (10.52, 21.22)	0.90
PD-1^-^CD8^+^ T cells (%)							
Naive	23.08 (15.84, 30.31)	12.80 (8.17, 17.43)		<0.001	16.93 (11.37, 22.49)	16.65 (9.03, 24.27)	0.85
Tcm	12.95 (9.16, 16.74)	15.06 (11.77, 18.34)		0.16	12.39 (7.54, 17.24)	18.84 (12.85, 24.84)	<0.01
Tem	38.73 (28.83, 48.63)	52.13 (43.63, 60.64)		<0.001	34.32 (26.47, 42.16)	34.51 (26.97, 42.05)	0.93
TEMRA	25.26 (17.79, 32.74)	20.01 (13.34, 26.68)		0.02	36.35 (26.56, 46.15)	30.00 (20.97, 39.03)	0.03

Paired data available at baseline and on-treatment were included. Date was shown as mean (lower 95% CI of mean, upper 95% CI of mean).

### Dynamic differentiation status of PD-1^+^ and PD-1^-^ CD8^+^ T-cell subsets affected by radiotherapy plus immunotherapy or CCRT treatment

**Figure 5:**
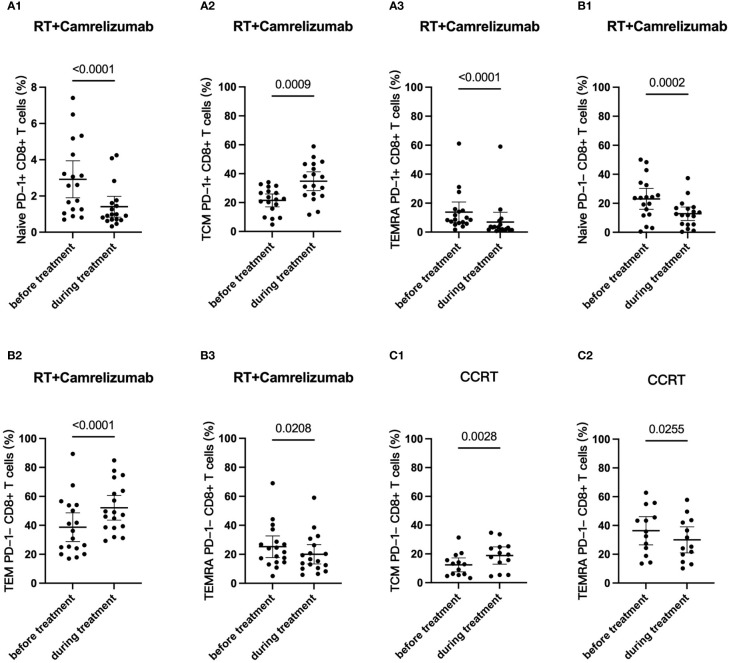
Dynamic differentiation status of PD-1^+^ and PD-1^-^ CD8^+^ T-cell subsets. **(A)** PD-1^+^CD8^+^ T cells in RT plus immunotherapy group. **(B)** PD1^-^CD8^+^ T cells in RT plus immunotherapy group. **(C)** PD1^-^CD8^+^ T cells in CCRT group.

We then longitudinally detected the differentiation status of PD-1^+^ and PD-1^-^ CD8^+^ T cells under radiotherapy plus immunotherapy or CCRT treatment (**Table 4**). We found that the percentage of Naïve T cells and TEMRA subsets reduced both in PD-1^+^ and PD-1^-^ CD8^+^ T cells while Tcm subset in PD-1^+^CD8^+^ T cells and Tem subset in PD-1^-^CD8^+^ T cells elevated significantly after RT plus camrelizumab treatment (**Figures 5A, B**; **Figures S5A, B**). The percentages of the four differentiation subsets in PD-1^+^CD8^+^ T cells did not change significantly after CCRT treatment (**Figure S5C**). The proportion of Tcm subset was elevated and TEMRA subset was reduced in PD-1^-^CD8^+^ T cells after CCRT treatment (**Figures 5C**; **Figure S5D**). These results suggested that RT plus camrelizumab treatment influenced the differentiation status of both PD-1^+^ and PD-1^-^ CD8^+^ T-cell subsets more deeply compared with CCRT treatment.

### Prognosis of patients undergoing radiotherapy combined with immunotherapy or CCRT treatment

**Figure 6:**
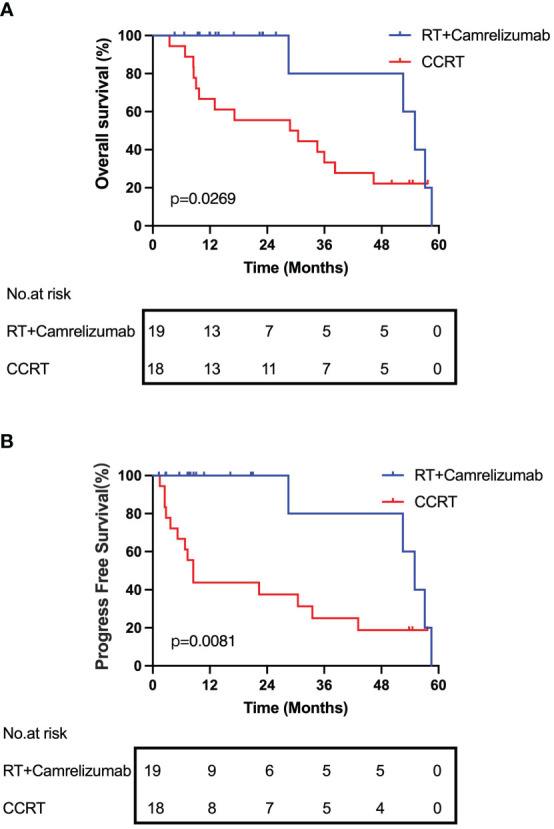
Prognosis of patients receiving RT plus camrelizumab or CCRT treatment. **(A)** OS. **(B)** PFS.

Lastly, we followed up and compared the survival of the patients between the RT plus camrelizumab group and CCRT group. The median OS and PFS were 54.97 and 54.97 months respectively in RT plus camrelizumab group and 29.62 and 8.52 months respectively in CCRT group. We found that the patients in RT plus camrelizumab group had both better OS and PFS compared with those in CCRT group (**Figure 6**). We did not find significant differences of survival in PD-1^+^ or PD-1^-^ CD8^+^ T cells in the individual treatment group, which was probably because of the limited number of patients included in the present study.

## Discussion

Our study revealed that PD-1^+^CD8^+^ T cells exhibited higher functional and activated characteristics and had more memory properties than PD-1^-^CD8^+^ T cells in the two treatment groups. Besides, both RT combined with immunotherapy and CCRT treatment activated PD-1^+^ and PD-1^-^ CD8^+^ T cells, while the two treatments had different effects on the differentiation state of PD-1^+^ and PD-1^-^ CD8^+^ T cells.

Both RT plus camrelizumab and CCRT treatments increased the expression levels of CD38 and HLA-DR in peripheral CD8^+^ T cells suggesting that both treatments activate CD8^+^ T cells. Besides, we found effective binding of anti-PD-1 antibody camrelizumab with PD-1 on CD8^+^ T cells during RT plus camrelizumab treatment. Anti-PD-1 antibody directly competed with PD-L1 on tumor cells to bind PD-1 on immune cells, thereby reducing the inhibitory effects of tumor cells on immune cells. This might be one of the reasons for the significantly better prognosis of radiotherapy combined with immunotherapy than CCRT in our preliminary survival analysis.

We found that functional molecules IFN-γ, as well as activated molecules CD38 and HLA-DR, were all higher in PD-1^+^CD8^+^ T cells than those in PD-1^-^CD8^+^ T cells in two groups both before and during treatment. These results indicated that PD-1^+^CD8^+^ T cells might have higher activity and stronger immune function than PD-1^-^CD8^+^ T cells. It was consistent with the reports that adoptive transfer of PD-1^+^ T cells to tumor-bearing mice resulted in tumor control, in contrast to the adoptive transfer of PD-1^-^ T cells (25, 26). Additionally, the ability of PD-1^+^CD8^+^ T cells maintained stable, regardless RT plus camrelizumab or CCRT administrated.

In addition, PD-1^+^CD8^+^ T cells consisted of less naïve but more memory subsets compared with PD-1^-^CD8^+^ T cells before treatment in both groups. It has been reported that expression of PD-1 identified a neoantigen-specific anti-tumor T cell response in peripheral CD8^+^ T cells (27). Neoantigen burdened by tumor cells stimulated and developed activation and differentiation of T cells resulting in more memory participants in the T-cell pool. The change of T-cell differentiation state was regularly accompanied by the change of activation and function characteristics. These evidences were consistent with our findings of higher proportions of memory subsets and stronger activation in PD-1^+^CD8^+^ T cells. Whether PD-1^+^CD8^+^ T cells contain more neoantigen-specific T cells with increased activation in ESCC deserved further exploration.

When dynamically monitoring the differentiation status of peripheral CD8^+^ T cells, Tcm subset increased in PD-1^+^CD8^+^ T cells and Tem subset increased in PD-1^-^CD8^+^ T cells after RT plus camrelizumab treatment, while only Tcm subset in PD-1^-^CD8^+^ T cells elevated after CCRT treatment. Since Tcm and Tem had different capabilities and life cycles responding to immune induction (28–30), our results suggested that RT plus camrelizumab and CCRT treatment might have partial distinct effects on the activation of memory T cells. The increase of different memory subsets in PD-1^+^ and PD-1^-^ CD8^+^ T cells might be related to the different expression of transcriptional factors in different memory subsets. It was demonstrated that T-bet, Blimp1, ID2, and STAT4 were associated with Tem subset, while Eomes, TCF1, BCL-6, ID3, and STAT3 were associated with Tcm subset (28). Our data showed that PD1^+^CD8^+^ T cells had higher expression level of Eomes than PD1^-^CD8^+^ T cells in RT plus camrelizumab group. This might be one of the reasons why Tcm subset increased in PD-1^+^CD8^+^ T cells rather than in PD-1^-^CD8^+^ T cells after RT plus camrelizumab treatment.

It was reported that PD-1^high^ CD8^+^ T cells predicted response to PD-1 blockade and correlated with increased overall survival and 80% of patients with clinical benefit exhibited PD-1^+^ CD8^+^ T-cell responses to PD-1 targeted immunotherapy in non-small cell lung cancer (31, 32). And tumor-specific CD8^+^ T cells were enriched among PD-1^+^ cells (27, 33). So our results that RT plus camrelizumab treatment increased PD-1^+^CD8^+^ Tcm while CCRT had no influence on any PD-1^+^CD8^+^ subset suggested that RT plus camrelizumab treatment might exhibit stronger CD8^+^ T-cell response than CCRT. This might also be one of the reasons for the better prognosis of radiotherapy combined with immunotherapy than CCRT in our preliminary survival analysis.

Radiotherapy promoted the release of tumor neoantigen, consequently activating neoantigen presentation and inducing specific anti-tumor immune response in local tumor microenvironment (34). Our present results from ESCC patients revealed that besides the local anti-tumor microenvironment, the systematic anti-tumor immune was also greatly promoted by these treatments.

For patients with locally advanced ESCC who were intolerable or refused surgery, CCRT is the standard treatment. However, the survival for these patients was still poor after CCRT. Recently, accumulated evidences have shown the important role of tumor-infiltrating immune cells in treatment outcome in ESCC (35, 36). The results from phase 3 clinical trials revealed the promising anti-tumor effect of anti-PD-1 antibody in advanced ESCC patients (37, 38). It is great interesting to find out whether combining chemoradiotherapy or radiotherapy with immunotherapy might increase the survival of ESCC patients. As a result, we laughed the first phase 1b clinical trial (No. NCT03222440) in 2017 to evaluate the toxicity and preliminary anti-tumor efficiency of radiotherapy plus camrelizumab in patients with locally advanced ESCC who were intolerable or refused surgery as well as CCRT. In the present study, we originally reported the systematic immune traits, including the activated, functional and differentiation status, under RT plus camrelizumab treatment and compared these findings with what we found in CCRT group. Our results would indicate the underling anti-tumor mechanisms under radiotherapy combined with immunotherapy.

Nevertheless, there were obvious limitations in this study. Because of the characteristics of the phase 1b clinical trial combing RT and camrelizumab, only 19 patients were finally included in the study. And the peripheral blood was also collected from patients receiving CCRT during the same period of the phase 1b study. The sample size was insufficient, and therefore our results were preliminary at present. We would continue to carry out relevant studies and collect more cases for analysis to verify our findings. In addition, we would take further studies on the underlying mechanisms of the different effects of the two treatments on the immune status of ESCC patients.

## Conclusions

This study identified the systematic immune status of patients undergoing treatment. The effects of RT concurrently combined with immunotherapy and CCRT on the expression levels of molecular markers of peripheral CD8^+^ T cells and on the characteristics and differentiation state of PD-1^+^ and PD-1^-^ CD8^+^ T-cell subsets were both similar and different. The two treatments had similar activation effects on peripheral CD8^+^ T cells with different PD-1 properties but had different effects on their differentiation status. In general, RT concurrently combined with immunotherapy attained better prognosis than CCRT, which had the potential to be an ideal option for patients with locally advanced ESCC. Our results provided potential clues to the reasons underlying the difference in prognosis of the two treatments. Indeed, our results are still preliminary and further researches are needed to verify our findings and clarify the underlying mechanisms.

## Data availability statement

The original contributions presented in the study are included in the article/supplementary material. Further inquiries can be directed to the corresponding authors.

## Ethics statement

The studies involving human participants were reviewed and approved by the Ethical Committee of Tianjin Medical University Cancer Institute and Hospital. The patients/participants provided their written informed consent to participate in this study.

## Author contributions

CY, QP, PW and WZ contributed to the study conception and design. ZG, XM, YangL, XW, DH, TZ, and XC contributed to samples preparation and data collection. Data analysis was performed by HW and YangL. The first draft of the manuscript was written by HW, YangL, CY and WZ, and polished by JZ. All authors read and approved the manuscript.

## Funding

This work was supported by the National Natural Science Foundation of China [grant number 82073348, 81602565 and 81872462], Chinese National Key Research and Development Project [2018YFC1315601] and Tianjin Key Medical Discipline (Specialty) Construction Project [TJYXZDXK-009A].

## Conflict of interest

Author JZ is employed by Jiangsu Hengrui Pharmaceuticals Co., Ltd.

The remaining authors declare that the research was conducted in the absence of any commercial or financial relationships that could be construed as a potential conflict of interest.

## Publisher’s note

All claims expressed in this article are solely those of the authors and do not necessarily represent those of their affiliated organizations, or those of the publisher, the editors and the reviewers. Any product that may be evaluated in this article, or claim that may be made by its manufacturer, is not guaranteed or endorsed by the publisher.

## Supplementary material

Figure S1 The expression levels of molecular markers in peripheral CD8+ T cells. (A) Eomes, T-bet and IFN-γ expressions in CD8+ T cells in RT plus camrelizumab group. (B) PD-1, Eomes and IFN-γ expressions in CD8+ T cells in CCRT group.

Figure S2 The expression levels of molecular markers in PD-1+ and PD-1- CD8+ T cells. (A) Schematics of the data processing of panel 1 and 2. (B) T-bet expression before RT plus immunotherapy treatment. (C) T-bet expression during RT plus immunotherapy treatment. (D) Eomes and T-bet expressions before CCRT treatment. (E) Eomes and T-bet expressions during CCRT treatment.

Figure S3 Dynamic expressions of Eomes, T-bet and IFN-γ in PD-1+ and PD-1- CD8+ T-cell subsets. (A) PD-1+CD8+ T cells in RT plus immunotherapy group. (B) PD-1-CD8+ T cells in RT plus immunotherapy group. (C) PD1+CD8+ T cells in CCRT group. (D) PD1-CD8+ T cells in CCRT group.

Figure S4 Differentiation subsets of memory T cells in PD-1+ and PD-1- CD8+ T cells. (A) Tem percentage during RT plus immunotherapy treatment. (B) Tcm percentage during CCRT treatment.

Figure S5 Dynamic differentiation status of PD-1+ and PD-1- CD8+ T-cell subsets. (A) PD-1+CD8+ T cells in RT plus immunotherapy group. (B) PD-1-CD8+ T cells in RT plus immunotherapy group. (C) PD-1+CD8+ T cells in CCRT group. (D) PD-1-CD8+ T cells in CCRT group.

## Abbreviations

AJCC, American Joint Committee on Cancer; CI, confidence interval; CCRT, concurrent chemoradiotherapy; ESCC, esophageal squamous cell carcinoma; NS, no significant; OS, overall survival; PFS, progress free survival; PBMCs, peripheral blood mononuclear cells; PBLs, peripheral blood lymphocytes; RT, radiotherapy; Tcm, central memory T cell; Tem, effector memory T cell; TEMRA, CD45RA+ effector memory T cell.
