# Supplementary figures and images for: Comparison of dynamic changes in the peripheral CD8+ T cells function and differentiation in ESCC patients treated with radiotherapy combined with anti-PD-1 antibody or concurrent chemoradiotherapy
Source: Front Immunol. 2022 Nov 21;13:1060695. doi: 10.3389/fimmu.2022.1060695 (PMC9720318; doi:10.3389/fimmu.2022.1060695)

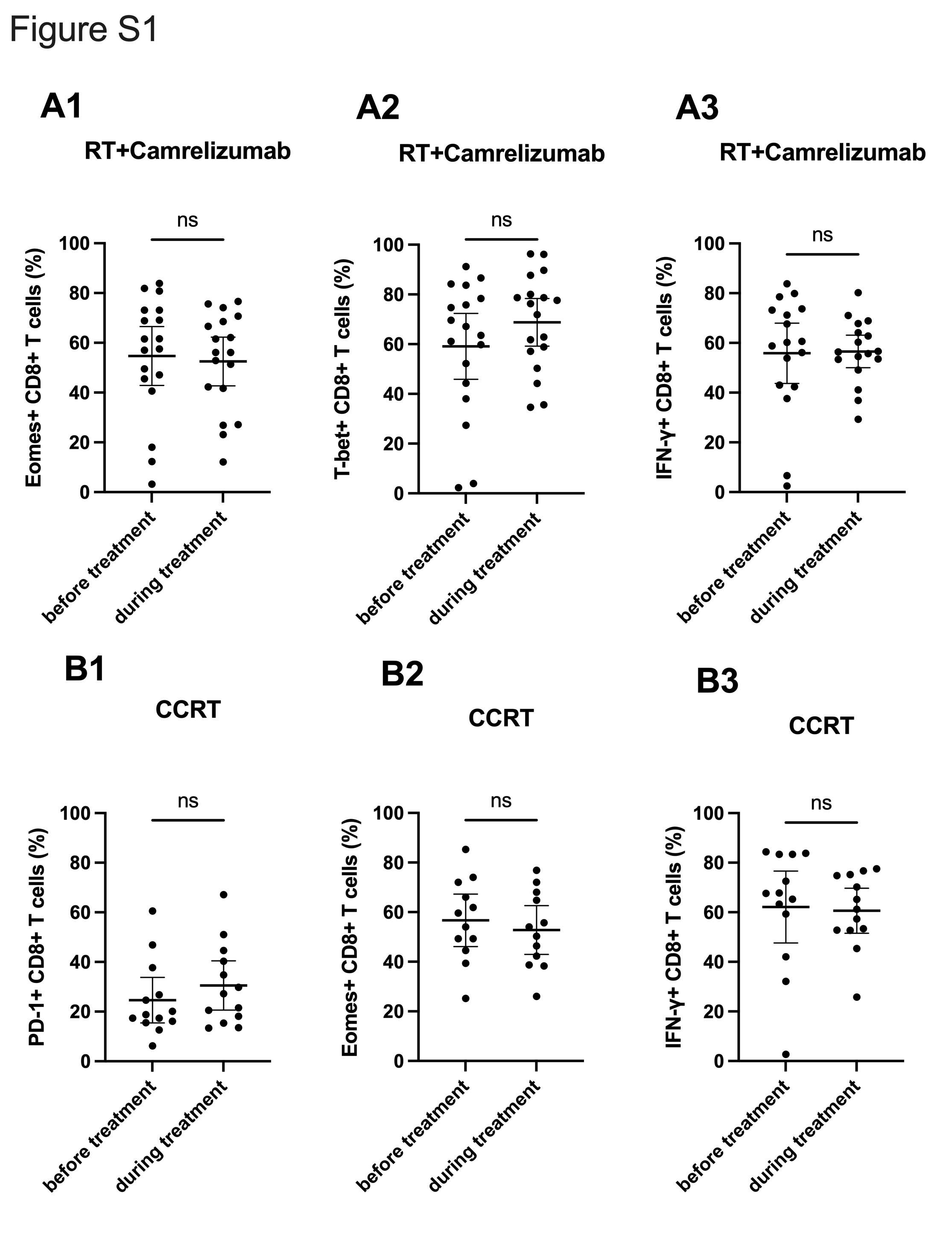

Supplement: Figure S1 — The expression levels of molecular markers in peripheral CD8+ T cells. (A) Eomes, T-bet and IFN-γ expressions in CD8+ T cells in RT plus camrelizumab group. (B) PD-1, Eomes and IFN-γ expressions in CD8+ T cells in CCRT group. [file Image_1.jpeg]

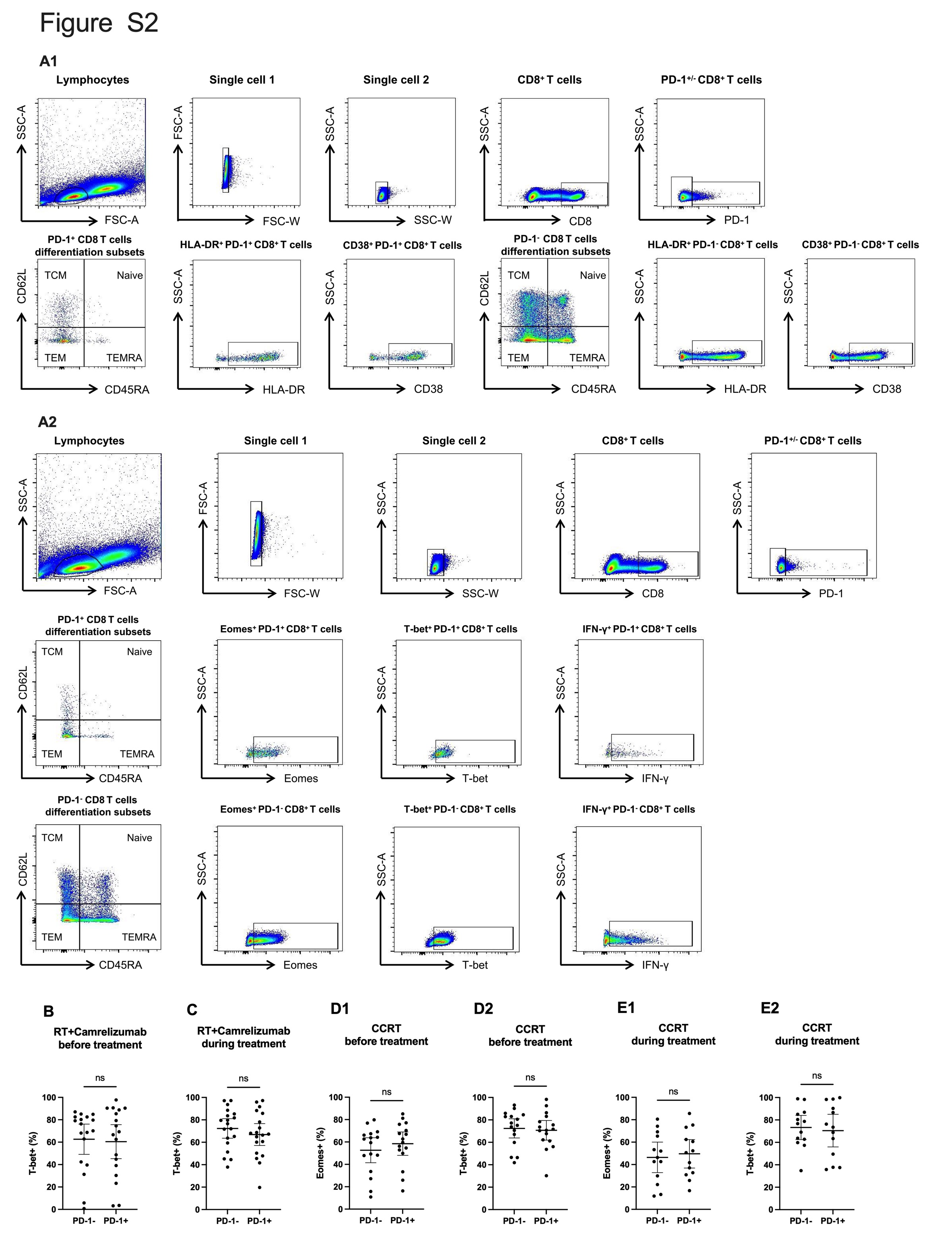

Supplement: Figure S2 — The expression levels of molecular markers in PD-1+ and PD-1- CD8+ T cells. (A) Schematics of the data processing of panel 1 and 2. (B) T-bet expression before RT plus immunotherapy treatment. (C) T-bet expression during RT plus immunotherapy treatment. (D) Eomes and T-bet expressions before CCRT treatment. (E) Eomes and T-bet expressions during CCRT treatment. [file Image_2.jpeg]

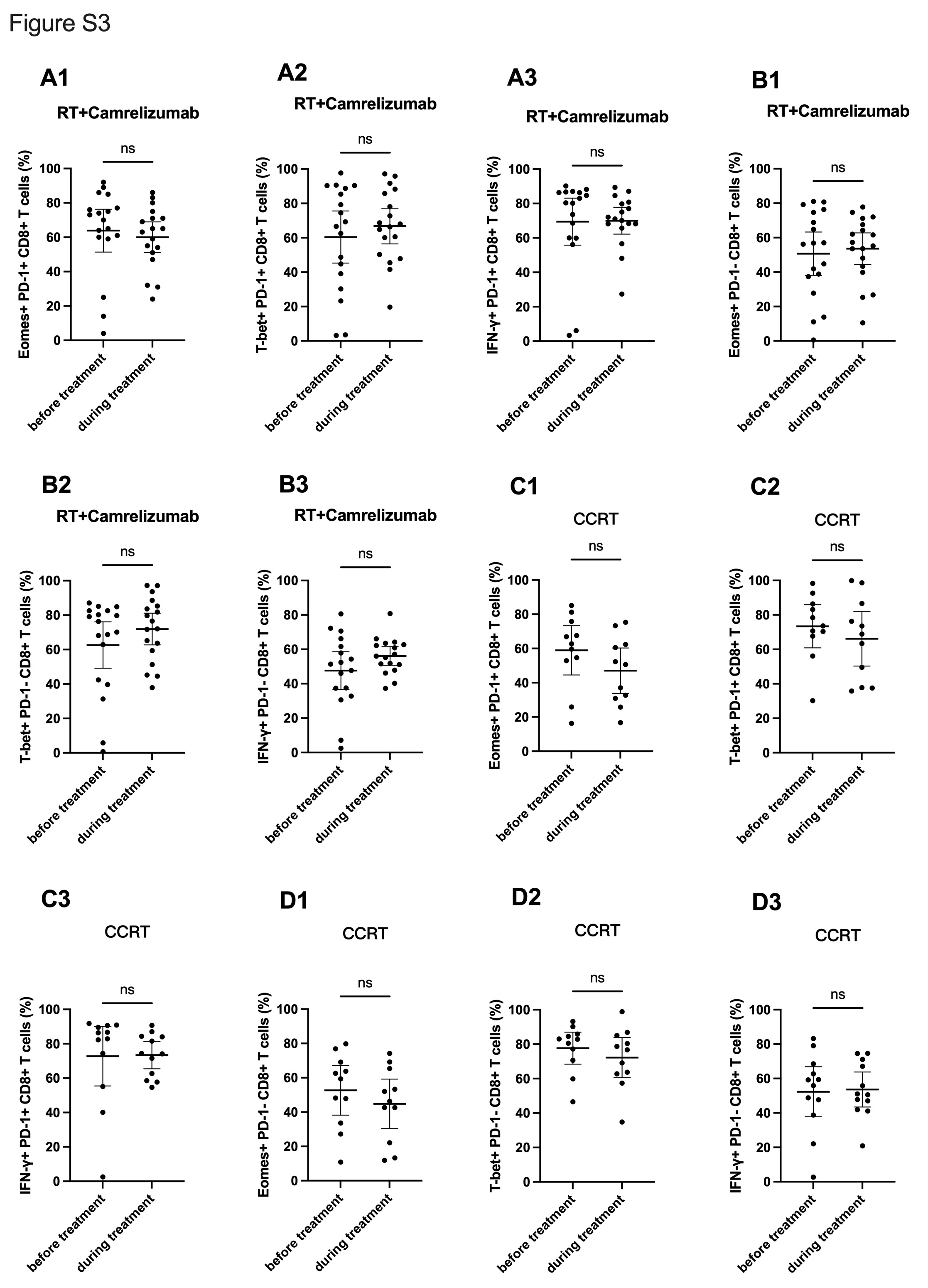

Supplement: Figure S3 — Dynamic expressions of Eomes, T-bet and IFN-γ in PD-1+ and PD-1- CD8+ T-cell subsets. (A) PD-1+CD8+ T cells in RT plus immunotherapy group. (B) PD-1-CD8+ T cells in RT plus immunotherapy group. (C) PD1+CD8+ T cells in CCRT group. (D) PD1-CD8+ T cells in CCRT group. [file Image_3.jpeg]

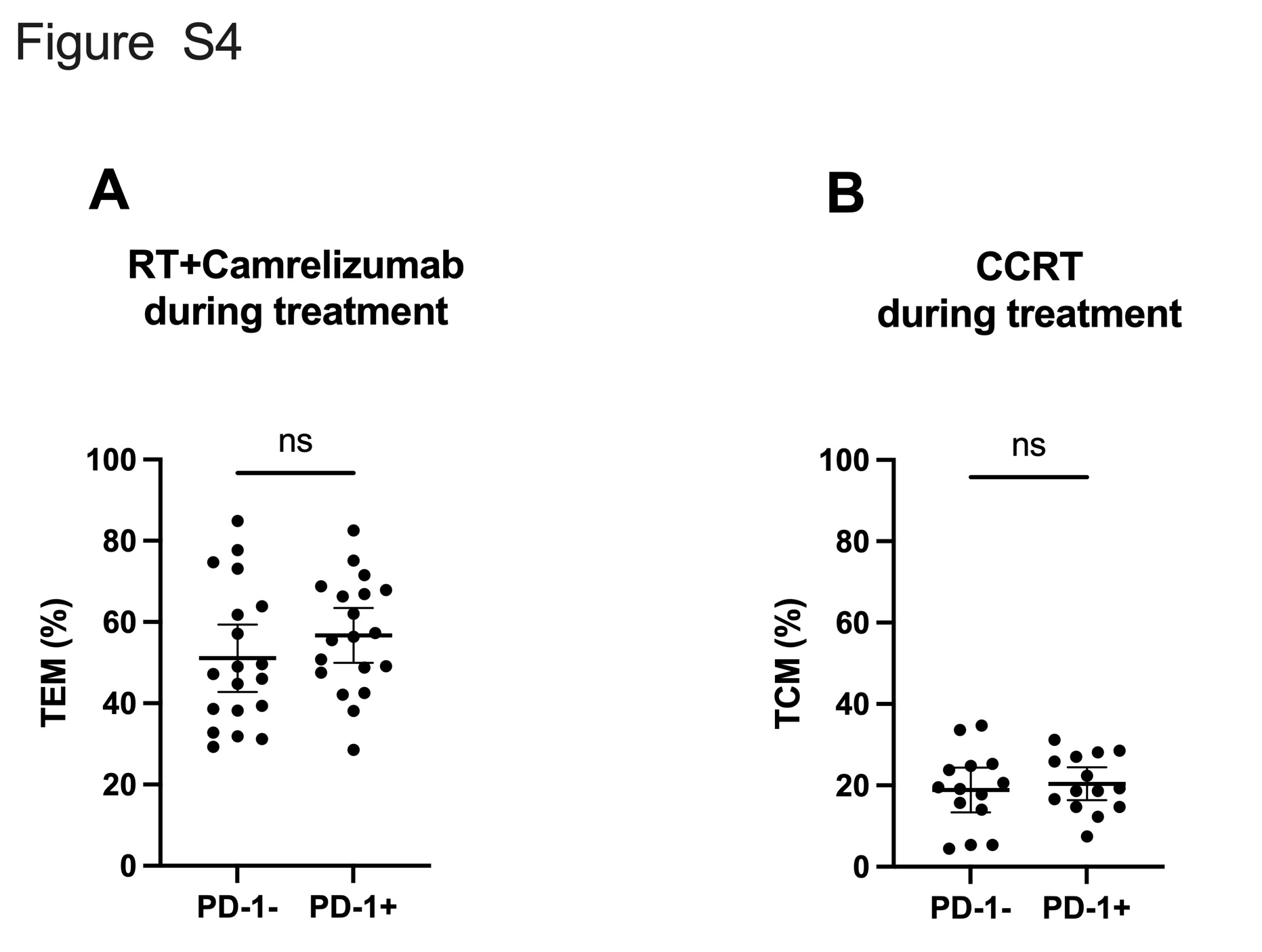

Supplement: Figure S4 — Differentiation subsets of memory T cells in PD-1+ and PD-1- CD8+ T cells. (A) Tem percentage during RT plus immunotherapy treatment. (B) Tcm percentage during CCRT treatment. [file Image_4.jpeg]

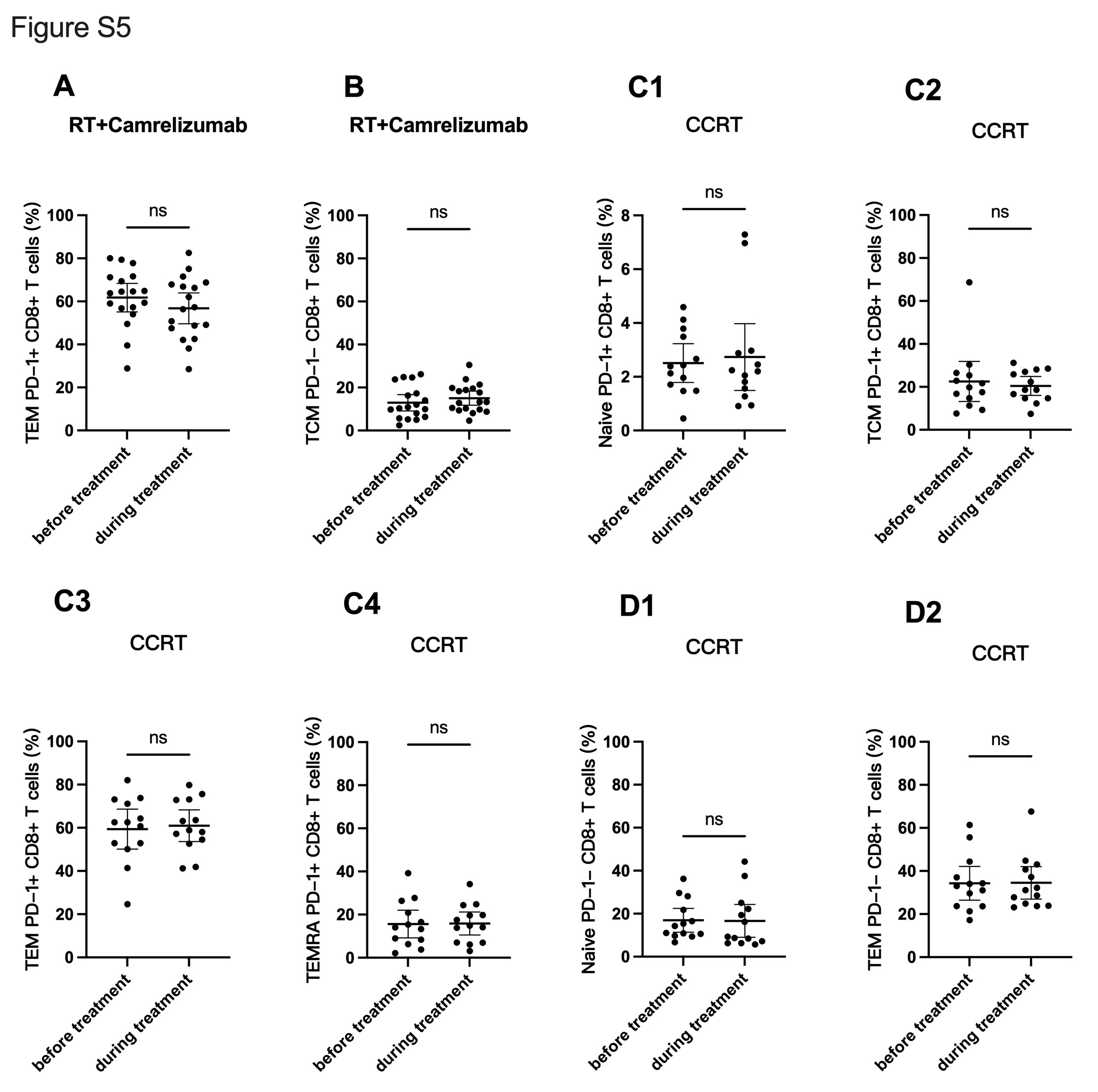

Supplement: Figure S5 — Dynamic differentiation status of PD-1+ and PD-1- CD8+ T-cell subsets. (A) PD-1+CD8+ T cells in RT plus immunotherapy group. (B) PD-1-CD8+ T cells in RT plus immunotherapy group. (C) PD-1+CD8+ T cells in CCRT group. (D) PD-1-CD8+ T cells in CCRT group. [file Image_5.jpeg]
